# ChAdOx1 NiV vaccination protects against lethal Nipah Bangladesh virus infection in African green monkeys

**DOI:** 10.1038/s41541-022-00592-9

**Published:** 2022-12-21

**Authors:** Neeltje van Doremalen, Victoria A. Avanzato, Kerry Goldin, Friederike Feldmann, Jonathan E. Schulz, Elaine Haddock, Atsushi Okumura, Jamie Lovaglio, Patrick W. Hanley, Kathleen Cordova, Greg Saturday, Emmie de Wit, Teresa Lambe, Sarah C. Gilbert, Vincent J. Munster

**Affiliations:** 1 Laboratory of Virology, National Institute of Allergy and Infectious Diseases, National Institutes of Health, Hamilton, MT USA; 2 Rocky Mountain Veterinary Branch, National Institute of Allergy and Infectious Diseases, National Institutes of Health, Hamilton, MT USA; 3 Oxford Vaccine Group, Department of Paediatrics, University of Oxford, Oxford, UK; 4 Chinese Academy of Medical Science (CAMS) Oxford Institute (COI), University of Oxford, Oxford, UK; 5 Pandemic Sciences Institute, Nuffield Department of Medicine, University of Oxford, Oxford, UK; 6 Present Address: Emory University School of Medicine, Atlanta, GA 30322 Georgia

**Keywords:** Live attenuated vaccines, Viral pathogenesis

## Abstract

Nipah virus (NiV) is a highly pathogenic and re-emerging virus, which causes sporadic but severe infections in humans. Currently, no vaccines against NiV have been approved. We previously showed that ChAdOx1 NiV provides full protection against a lethal challenge with NiV Bangladesh (NiV-B) in hamsters. Here, we investigated the efficacy of ChAdOx1 NiV in the lethal African green monkey (AGM) NiV challenge model. AGMs were vaccinated either 4 weeks before challenge (prime vaccination), or 8 and 4 weeks before challenge with ChAdOx1 NiV (prime-boost vaccination). A robust humoral and cellular response was detected starting 14 days post-initial vaccination. Upon challenge, control animals displayed a variety of signs and had to be euthanized between 5 and 7 days post inoculation. In contrast, vaccinated animals showed no signs of disease, and we were unable to detect infectious virus in tissues and all but one swab. No to limited antibodies against fusion protein or nucleoprotein antigen could be detected 42 days post challenge, suggesting that vaccination induced a very robust protective immune response preventing extensive virus replication.

## Introduction

Nipah virus (NiV) is a highly pathogenic re-emerging member of the *Paramyxovirus* family, genus *Henipavirus*. NiV causes sporadic infections in humans, resulting in severe neurological and respiratory disease, often with a fatal outcome. NiV was first detected in 1998, when the genotype NiV-Malaysia (NiV-M) caused an outbreak of severe encephalitis in pig farmers from Malaysia and abattoir workers in Singapore, with a case-fatality rate of 38%^1^. Since 2001, outbreaks with NiV-Bangladesh (NiV-B) have occurred almost yearly in Bangladesh^2^, resulting in 319 accumulated cases and 225 associated deaths (case-fatality rate 71%)^3^. Outbreaks have also been reported in India^4^.

Although the total number of cases caused by NiV are limited, the virus causes severe disease, transmits between humans^5,6^, and can infect a wide range of animals^7^; thus, NiV is categorized by the WHO as a pathogen with epidemic potential which poses a great public health risk and requires research aimed at the development of countermeasures^8^.

Several vaccine candidates have been evaluated in animal models^9^. One vaccine is based on the glycoprotein of Hendra virus (HeV), another member of the genus *Henipavirus*^10^. HeV-sG, a soluble form of the HeV receptor binding glycoprotein, was marketed by Zoetis, Inc., in Australia as an equine vaccine against HeV under the name Equivac^®^ HeV. It was the first commercialized vaccine against a BSL4 agent^11^. Recently, it was shown that HeV-sG vaccination can protect African green monkeys (AGMs) against lethal NiV disease as early as 7 days post immunization^11^.

In the current study, we are testing efficacy of a different NiV vaccine candidate in AGMs. ChAdOx1 is a replication-deficient simian adenoviral vector, which has been developed for a multitude of different pathogens by the University of Oxford. A vaccine based on this vector named ChAdOx1 nCoV-19 (also known as AZD1222, Vaxzevria, or Covishield) has been developed against severe acute respiratory syndrome coronavirus 2 (SARS-CoV-2), the etiological agent of COVID-19. The efficacy of ChAdOx1 nCoV-19 has been shown in several randomized controlled clinical trials^12–14^. Furthermore, more than 2.5 billion doses of ChAdOx1 nCoV-19 vaccinations have been distributed to more than 170 countries, and it is the most widely used vaccine in the Covax program, which focuses on global equitable access to COVID-19 vaccines.

We have previously demonstrated that a single dose of ChAdOx1 NiV, which encodes the receptor binding protein (G) of NiV-B, fully protected Syrian hamsters against a lethal dose of NiV-B or NiV-M^15^. Here, we show that a single dose of ChAdOx1 NiV results in a robust innate and adaptive immune response and is fully protective against lethal disease in AGMs. Furthermore, no to limited immune responses against nucleoprotein or fusion protein were detected in vaccinated animals post challenge, but were detected in control challenged animals, suggesting that vaccination provides near complete protection against NiV infection.

## Results

### ChAdOx1 NiV vaccination of African green monkeys elicits a potent adaptive immune response

**Fig. 1:**
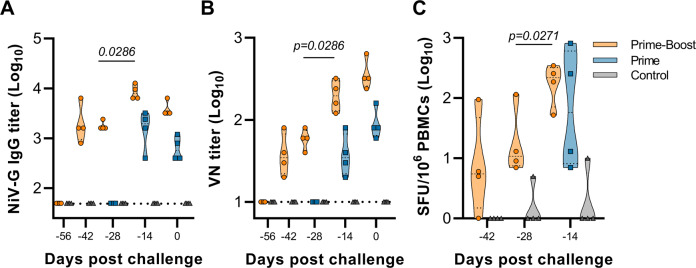
Vaccination with ChAdOx1 NiV in AGMs induces humoral and cellular immune responses. **A** Truncated violin plot shows evidence of NiV-G-specific IgG in serum at the indicated days post challenge in animals receiving intramuscular ChAdOx1 NiV via a prime-boost (orange, *N* = 4) or prime-only regimen (blue, *N* = 4), or ChAdOx1 GFP via a prime-boost regimen (gray, *N* = 4). **B** Truncated violin plot of neutralizing antibodies in serum are shown. **C** Truncated violin plot of NiV-G protein-specific T-cell responses in PBMCs isolated from vaccinated or controls animals at indicated time points minus −56 days post-challenge response. SFU spot-forming units. Black lines indicate median; dotted lines in violin plots indicate quartiles. The dotted line shows the limit of detection.

Four animals per group were vaccinated via the intramuscular (I.M.) route with ChAdOx1 NiV using a prime-boost regimen at 56 and 28 days before challenge (animals 1–4), or a prime-only regimen at 28 days before challenge (animals 5–8). As a control, four animals were vaccinated via the I.M. route with ChAdOx1 GFP at 56 and 28 days before challenge (animals 9–12). Binding antibody titers against NiV-G protein were determined at day of vaccination (−56 and −28 days post inoculation (DPI)), 14 days post vaccination (−42 and −14 DPI), and day of challenge (0 DPI). NiV-G-specific IgG antibodies could be detected as early as 14 days post vaccination and were significantly increased upon boost vaccination (Two-tailed Mann–Whitney test, *p* = 0.0286). All vaccinated animals had detectable NiV-G-specific antibodies on the day of challenge (Fig. 1A). No NiV-G-specific IgG antibodies were detected in control animals. Likewise, virus neutralizing antibodies were detected in all vaccinated animals at day of challenge and were significantly increased upon boost vaccination (Fig. 1B, Mann–Whitney test, *p* = 0.0286). NiV-G-specific T-cell responses were investigated using a peptide library divided into six peptide pools that spanned the full length of NiV-G. A single vaccination resulted in specific T-cell responses in all animals, and a subsequent boost vaccination raised T-cell responses significantly above control T-cell responses (Fig. 1C, Mann–Whitney test, *p* = 0.0271).

### Vaccinated animals do not show signs of disease

**Fig. 2:**
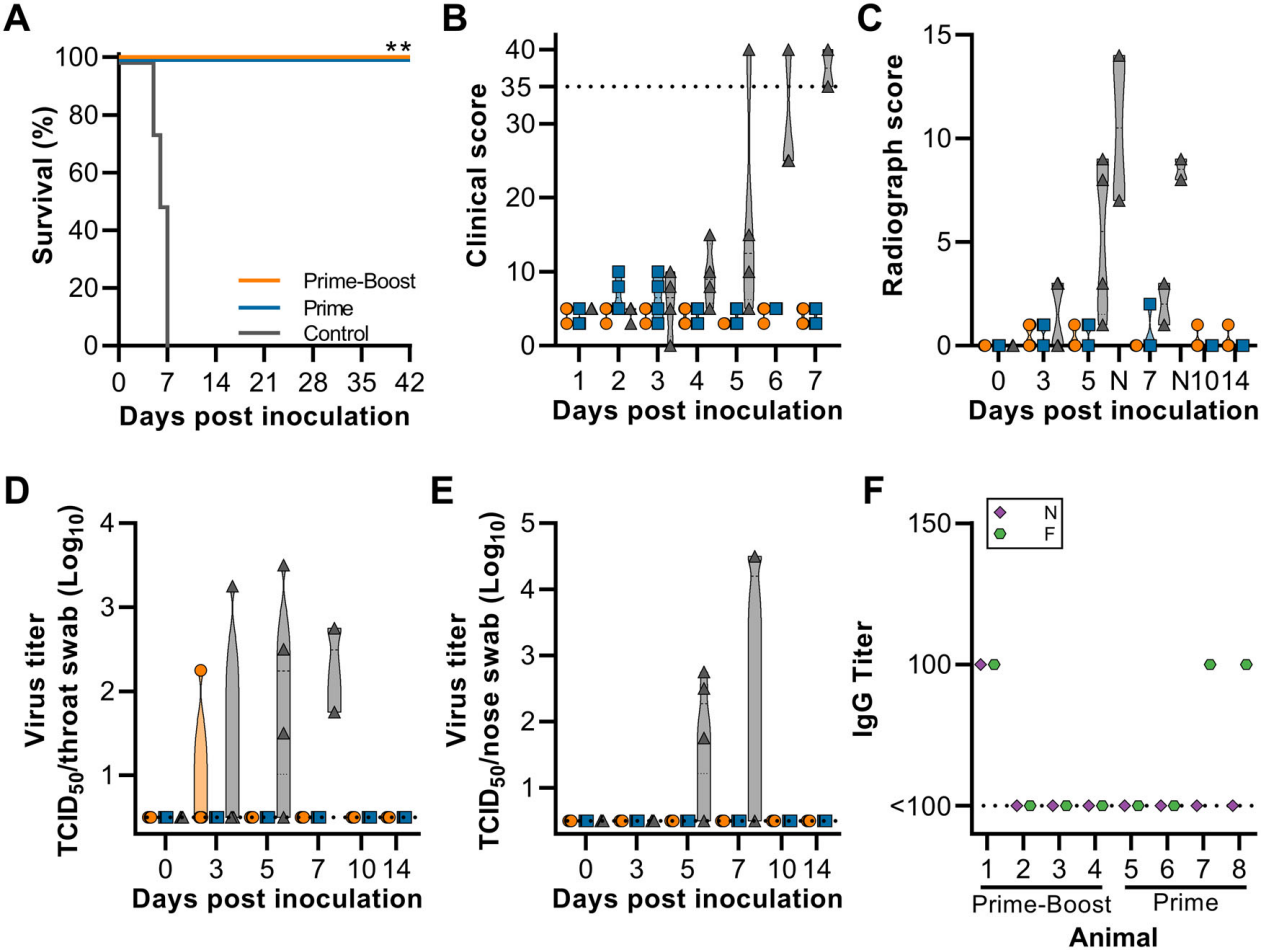
Clinical signs and NiV detection in vaccinated AGMs upon virus challenge. 28 days post final vaccination, AGMs (*N* = 4 per group) were inoculated with NiV-B and monitored for up to 42 days. **A** Survival of AGMs. **B** Truncated violin plot of daily clinical score. Dotted line indicates humane endpoint score requiring euthanasia. **C** Truncated violin plot of thoracic radiograph scores on exam days and day of euthanasia (N, controls only). **D**, **E** Truncated violin plots of infectious NiV in throat swabs (**D**) and nose swabs (**E**). At 7 DPI, only 2 control animals were part of the study. Dotted line indicates limit of detection. **F** Truncated violin plot shows no or very low (1:100 dilution) evidence of NiV- nucleoprotein (N, purple diamond) or NiV-fusion glycoprotein (F, green octagon) -specific IgG in serum at 42 DPI. Dotted line indicates limit of detection. For all panels, orange indicates prime-boost vaccinated animals, blue indicates prime-only vaccinated animals, and gray indicates control animals.

Animals were inoculated via the intranasal and intratracheal route with 2 × 10^5^ TCID_50_ of NiV-B. Animals were checked daily for clinical signs. Animals vaccinated with ChAdOx1 GFP displayed a variety of signs starting at 3 DPI, including neurological signs and respiratory signs (Supplementary Table 1). All four animals in this group reached the humane endpoint clinical score of 35 or higher between 5 to 7 DPI and were euthanized. In contrast, no signs of disease were observed in animals vaccinated with ChAdOx1 NiV (Fig. 2A, B, Supplementary Table 1). Exams were performed on 0, 3, 5, 7, 10, 14, 21, 28, 35, and 42 DPI. Radiographs were scored as previously described^16^. Whereas no to limited changes from baseline were observed in ChAdOx1 NiV vaccinated animals (score between 0–2), radiograph scores of control-vaccinated animals started increasing at 3 DPI (score between 0–3), and continued to increase until day of euthanasia (score between 7–14, Fig. 2C). Throat and nose swabs were collected on all exam days and the presence of infectious virus was investigated. Infectious virus could be detected in both nose and throat swabs of three out of four control-vaccinated animals. In contrast, all swabs obtained from animals vaccinated with ChAdOx1 NiV were negative, except for one throat swab obtained at 3 DPI from one animal (animal 1) in the prime-boost group (Fig. 2D, E). These results were confirmed when the viral load in swabs was investigated: limited to no viral RNA was detected in most swabs obtained from vaccinated animals, whereas high viral load was detected in all swabs that were positive for infectious virus, including the throat swab of animal 1 on 3 DPI (Supplementary Fig. 1). The presence of binding antibodies against fusion glycoprotein (F) and nucleoprotein (N) of NiV was then investigated in sera obtained from vaccinated animals at 42 DPI. A low titer of binding antibodies against N was found in sera from animal 1 (which had a positive throat swab on 3 DPI), and antibodies against F were detected in sera from animal 1, 7, and 8 (Fig. 2F).

### No evidence of NiV detected in tissues of vaccinated animals

**Fig. 3:**
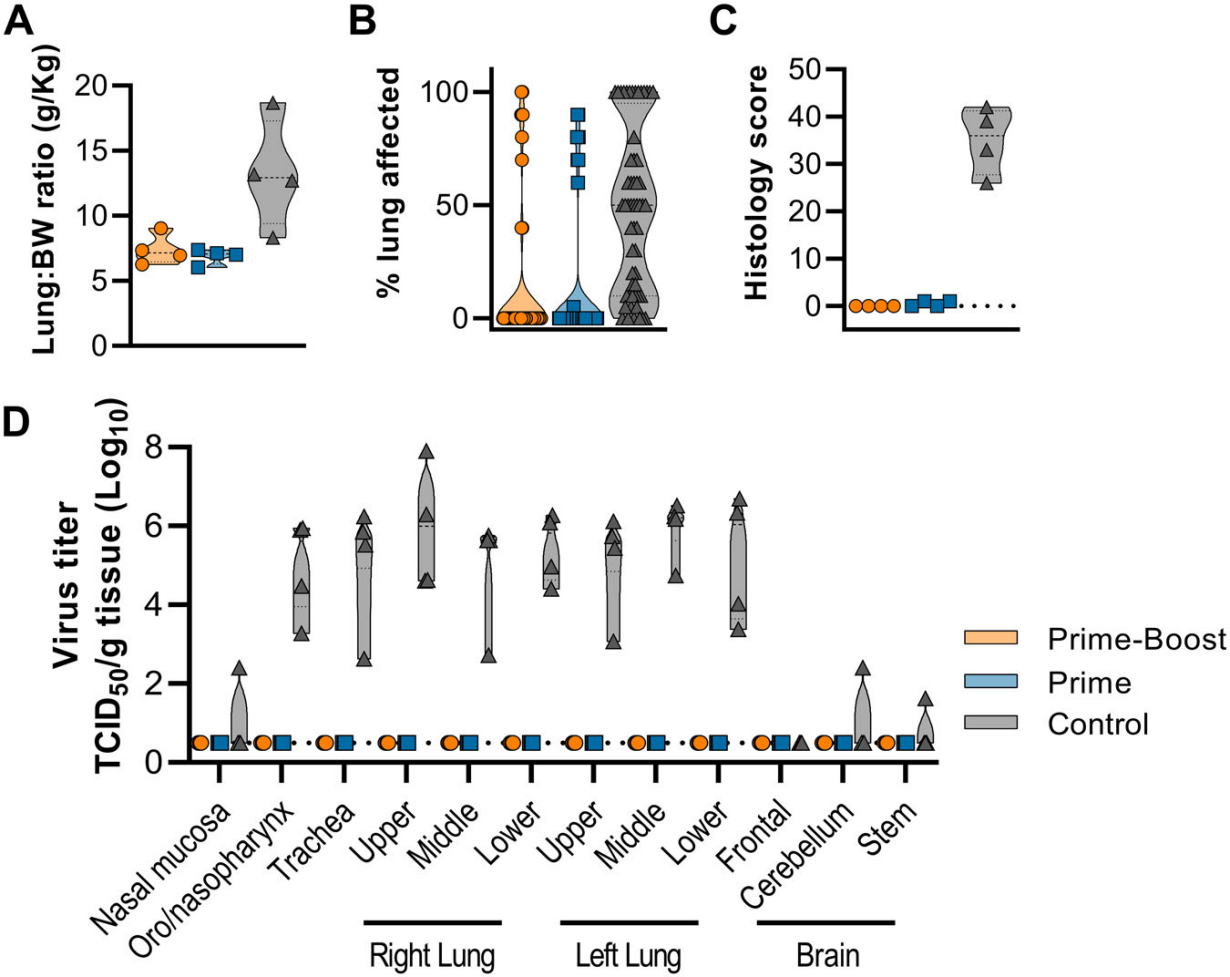
No evidence of NiV infection detected in vaccinated animals. AGMs were euthanized when a clinical score of 35 was reached (all control animals, 5–7 DPI) or on 42 DPI (all vaccinated animals). **A** Truncated violin plot of lung:body weight (BW) ratio. **B** Truncated violin plot of percentage of gross lung lesions. which were scored for each lung lobe, ventral and dorsal. **C** Truncated violin plot of histology score. All six lung lobes were scored and cumulative score is shown per animal (maximum score = 90). **D** Truncated violin plot of infectious virus detected in respiratory tract and brain tissue. For all panels, orange indicates prime-boost vaccinated animals, blue indicates prime-only vaccinated animals, and gray indicates control animals. No statistical tests were performed since samples were obtained on different days post challenge.

Animals were euthanized when euthanasia criteria were reached (control group, 5–7 DPI) or at 42 DPI (end of study, vaccinated animals). Lung:body weight ratio, indicative of lung edema, was higher for control animals than for vaccinated animals (Fig. 3A). At necropsy, the percentage of each lung lobe that showed gross lesions was scored and was found to be higher in the control animals (Fig. 3B). Lungs of control animals failed to collapse (3 out of 4), and pleural effusion was observed in all animals. Cervical lymph node enlargement and edematous mediastinal lymph nodes were observed in two and three out of four control animals, respectively. One control animal showed a diffuse hemorrhage from the medulla oblongata to the cervical spinal cord, with petechiae on the cerebellum.

**Fig. 4:**
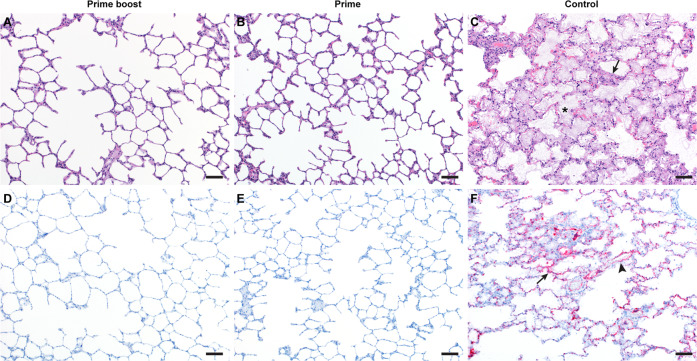
Pulmonary effects of ChAdOx1 NiV vaccine efficacy in the AGM model of NiV infection. **A**–**C** Lung tissue sections were stained with hematoxylin and eosin. **A**, **B** No pathology was observed. **C** Moderate to marked interstitial pneumonia with abundant fibrin (arrow) and edema (asterisk). **D**, **F** In situ hybridization for NiV RNA, resulting in a red stain. **D**, **E** No immunoreactivity was observed. **F** Immunoreactive vascular endothelium (arrowhead) and pneumocytes (arrow). Magnification: ×100; bar = 50 µm.

Histologically, no pulmonary pathology consistent with NiV lesions was observed in vaccinated animals (Fig. 4A, B), and consequently the histology score was low (Fig. 3C, Supplementary Table 1). Likewise, no NiV RNA staining was detected in lung tissue by in situ hybridization (Fig. 4D, E). In stark contrast, histological lesions were present in lung tissue obtained from all control animals and were characterized as multifocal, random, minimal (1–10%) to marked (51–75%) bronchointerstitial pneumonia. The pneumonia was characterized by thickening of alveolar septa by edema fluid and fibrin and small to moderate numbers of macrophages, syncytial cells, and neutrophils (Fig. 4C). In situ hybridization revealed abundant viral RNA distributed throughout lesions in vascular endothelium and type I and II pneumocytes in tissue from control animals (Fig. 4F).

Immunohistochemistry was performed on the brain using a polyclonal antibody against the entire N protein and in all animals, what was suspected to be spurious labeling in the trigeminal tracts of the brain stem was observed. In a subset of infected animals, in situ hybridization was performed using both positive and negative sense probes and no viral RNA was detected in the brain stem. The antibody was tested on noninfected African green monkeys, and the same pattern of immunoreactivity was observed in the brain stem. A different antibody developed against a specific peptide in the N protein was then used on noninfected animals and a subset of infected animals and no erroneous immunoreactivity was observed in the brain stem (Supplementary Fig. 2). Thus, no evidence of virus replication in the brain of any animals was detected. Furthermore, no antigen staining was detected in the lung tissues of the vaccinated animals but was scattered to numerous in the lungs of all the control animals.

Infectious virus was only detected in tissue obtained from control animals and not in tissue obtained from vaccinated animals, although it should be noted that different days post challenge are compared (Fig. 3D, Supplementary Fig. 3).

## Discussion

We evaluated the vaccine candidate ChAdOx1 NiV, which we previously showed to be fully protective in Syrian hamsters^15^, in the lethal AGM NiV challenge model. We show here that a single dose of ChAdOx1 NiV was fully protective against a lethal challenge with NiV-B in AGMs. Furthermore, we found very limited evidence of virus replication in vaccinated animals; all but one swab was negative for infectious virus, no virus was found in tissues obtained from vaccinated animals, and no to a very limited humoral immune response was detected against NiV-F or N proteins after challenge of vaccinated animals. These data suggest the vaccine prevents extensive replication of NiV in AGMs.

Bossart et al.^17^ show a similar lack of NiV-F-specific antibodies in vaccinated animals. AGMs were vaccinated with a HeV-G subunit vaccine 6 and 3 weeks before challenge with a lethal dose of NiV. Serum was obtained throughout the experiment and NiV-F-specific IgM could not be detected at any point. Furthermore, whereas vaccination induced high IgG titers against NiV-G and HeV-G, there was no increase in G-specific IgG titers after challenge with NiV. Together, these data suggest a lack or reduction of virus replication in vaccinated animals^17^. Likewise, in a study performed by Lo et al.^18^, hamsters were vaccinated with replication-deficient VSV-based NiV-G or NiV-F. Virus neutralizing (VN) antibody titers were measured in serum obtained at 28 days post vaccination and 32 days post challenge. No anamnestic response was detected in vaccinated animals, suggesting that this vaccine may also provide severely reduced virus replication^18^. In a study by Prescott et al., AGMs were vaccinated with rVSV-EBOV-GP-NiVG, 29 days before challenge with NiV Malaysia. An increase in binding and neutralizing antibodies titer between 0 DPI and 16/17 DPI (termination of study) was found in 2/3 vaccinated animals^19^.

The approval of a NiV vaccine is hindered by the feasibility of an efficacy trial due to the sporadic nature of infections, the large geographical area where the spillover occurs and the low number of annual cases^20^. To address these complications, the U.S. Food and Drug Administration implemented the ‘Animal Rule’ in 2002^21^. This rule can be utilized to establish efficacy based on studies performed in animal models that faithfully recapitulate human disease^20^. Thus far, 18 products have been approved via this route^22^. In both hamster and AGM NiV models, vaccination with ChAdOx1 NiV resulted in induction of high antibody titers coupled with complete protection against lethal NiV disease. ChAdOx1 NiV is based on the same vector as ChAdOx1 nCoV-19, which has been administered in more than 180 countries worldwide, and 2.5 billion doses have been delivered.

Anti-vector immunity may not play an important role; a third dose of ChAdOx1 nCoV-19 after a two-dose schedule of the vaccine was able to boost adaptive immune responses against SARS-CoV-2 to a higher level^23^ and is likely to have little effect on the administration of a different antigen expressed from the same vector. A Phase I clinical trial is needed to investigate the use of one or two doses in humans, as well as the effect of previous ChAdOx1 vector vaccinations. The ChAdOx1 nCoV-19 vaccine is currently produced by 25 facilities worldwide. The manufacturing protocol will be identical for other vaccines based on the same vector, making production of a ChAdOx1 NiV vaccine simple to implement and at a low cost of less than $5 per dose. Thus, safety profiles obtained in ChAdOx1 nCoV-19 clinical studies^12^ combined with ChAdOx1 NiV efficacy studies in animal models^15^ and Phase I/II clinical trials may provide sufficient information for approval of ChAdOx1 NiV.

We performed intensive investigation of virus invasion of brain tissue in the vaccinated and control animals. Since infection of the central nervous system (CNS)^24^ as well as late-onset and relapsed encephalitis have been documented in humans^25,26^, it is of the utmost importance to ensure that a NiV vaccine does not just prevent disease, but also prevents the virus from entering the CNS. We found no evidence of viral presence in brain tissue, as investigated via titrations and immunohistochemistry. It is possible that increasing the time to necropsy could have increased the chances of capturing a relapse, but due to time and space restrictions associated with BSL4 it was decided to monitor for clinical signs of encephalitis and perform a thorough histological evaluation of the AGM’s CNS on day 42, which are associated with neurological signs^27^. In this, no evidence of histologic lesions that could expand and cause neurological signs in the future, nor evidence of the presence of viral antigen were found in the CNS. Combined with the lack of clinical signs, we hypothesize that no relapse would have occurred in the animals at later time points.

We found one throat swab in vaccinated animal 1 on 3 DPI that was positive for infectious virus and viral RNA. This animal tested negative for infectious virus in its swabs and tissues later, although a low amount of viral RNA was detected in a throat swab taken on 7 DPI. The relevance of this finding is unclear since neither virus replication was found in the other animals nor did the animal show any signs of disease. Importantly, no evidence of viral encephalitis was found in this animal.

In the current manuscript, we show that both one and two doses of ChAdOx1 NiV are efficacious against disease in African green monkeys, arguing that even one dose may be sufficient for protection in humans. Although several NHP studies have successfully investigated the efficacy of NiV vaccines^17,19,28–30^, thus far only two vaccines have advanced into clinical trials^31,32^. Here, we show that the widely used ChAdOx1 vector can be modified to provide protection against NiV in a lethal NHP model. Previously, similar work investigating the efficacy of a ChAdOx1 MERS vaccine in rhesus macaques^33^ was instrumental in the development of the ChAdOx1 nCoV-19 vaccine^34^. If the next pandemic were to be caused by a member of the genus *Henipavirus*, the current study could be influential in the development of a rapid vaccine. Future studies, such as a Phase I clinical trial and further efficacy studies in AGMs, should aim to obtain approval via the FDA Animal Rule.

## Methods

### Ethics statement

The Institutional Animal Care and Use Committee (IACUC) at Rocky Mountain Laboratories approved all animal study requests, which were conducted in an Association for Assessment and Accreditation of Laboratory Animal Care (AAALAC)-accredited facility, following the basic principles and guidelines in the Guide for the Care and Use of Laboratory Animals 8th edition, the Animal Welfare Act, United States Department of Agriculture and the United States Public Health Service Policy on Humane Care and Use of Laboratory Animals.

Animals were kept in climate-controlled rooms with a fixed light/dark cycle (12 h/12 h). African green monkeys were housed in individual primate cages allowing social interactions, fed a commercial monkey chow, treats, and fruit with ad libitum water and were monitored at least twice daily. Environmental enrichment consisted of a variety of human interaction, commercial toys, videos, and music. The Institutional Biosafety Committee (IBC) approved work with infectious Nipah virus strains under BSL4 conditions. All sample inactivation was performed according to IBC approved standard operating procedures for removal of specimens from high containment^35,36^.

### Study design

Twelve African green monkeys (6 M, 6 F) between 3 and 5 years old were sorted by sex, then by weight, and then randomly divided into three groups of four animals. Group 1 was vaccinated with ChAdOx1 NiV at −56 and −28 DPI, group 2 was vaccinated with ChAdOx1 NiV at −28 DPI, group 3 was vaccinated with ChAdOx1 GFP at −56 and −28 DPI. All vaccinations were done intramuscularly with 2.5 × 10^10^ VP/animal diluted in sterile PBS. Animals were challenged with Nipah Bangladesh (AY988601) diluted in sterile DMEM at 0 DPI; all animals received 4 mL intratracheally (2.5 × 10^4^ TCID_50_/mL) and 1 mL intranasally (1 × 10^5^ TCID_50_/mL). Animals were scored daily by the same person who was blinded to study group allocations using a standardized scoring sheet^37^. Scoring was based on the following criteria: general appearance, skin and coat appearance, discharge, respiration, feces and urine appearance, appetite, and activity. Clinical exams were performed on −56, −42, −28, −14, 0, 3, 5, 7, 10, 14, 21, 28, 35, and 42 DPI. Blood, nasal, and throat swabs were collected on all exam dates, whilst animals were sedated with ketamine (10–15 mg/kg) or Telazol (3–6 mg/kg). Thoracic radiographs were obtained on clinical exam days prior to any other procedures with a Fuji Digital X-Ray System (Universal Imaging, Bedford Hills NY) utilizing standard thoracic technique charts. Radiographs were evaluated and scored for the presence of pulmonary infiltrates by clinical laboratory animal veterinarians who received appropriate training. The scoring^16^ was done as follows: each lung lobe (upper left, middle left, lower left, upper right, middle right, lower right) was scored individually based on the following criteria: 0 = normal examination; 1 = mild interstitial pulmonary infiltrates; 2 = moderate interstitial pulmonary infiltrates, perhaps with partial cardiac border effacement and small areas of pulmonary consolidation (alveolar patterns and air bronchograms); and 3 = pulmonary consolidation as the primary lung pathology, seen as a progression from grade 2 lung pathology. Day 0 radiographs are taken prior to inoculation, and thus serve as a baseline for each animal. All subsequent radiographs are compared to the Day 0 radiographs, evaluated for changes from baseline and scored based on the criteria noted above. At study completion, thoracic radiograph findings are reported as a single cumulative radiograph score for each animal on each exam day; scores may range from 0 to 18. Animals were euthanized on 42 DPI or when euthanasia criteria were reached in accordance with the AVMA Guidelines for Euthanasia. Specifically, animals were under deep anesthesia using either Ketamine (10–20 mg/kg) or Telazol (3–8 mg/kg) and then given a commercial euthanasia solution containing pentobarbital sodium and phenytoin solution either intravenously or intracardiac (1 ml/5 kg). Tissues were collected immediately after euthanasia.

### Generation of vaccine ChAdOx1 NiV

ChAdOx1 NiV^15^ was produced as follows: the G gene from Nipah virus (Bangladesh outbreak 2008–2010, Genbank accession number: JN808864.1) was codon optimized for humans, synthesized by GeneArt (Thermo Fisher Scientific), and cloned into a transgene expression plasmid comprising a modified human cytomegalovirus immediate early promoter (CMV promoter) with tetracycline operator (TetO) sites and the polyadenylation signal from bovine growth hormone (BGH). This expression cassette was inserted into the E1 locus of the genomic clone of ChAdOx1 using site-specific recombination^38^. The virus was rescued and propagated in T-REx-293 cells (Invitrogen). Purification was by CsCl gradient ultracentrifugation, virus titers were determined by hexon immunostaining assay and viral particles were calculated based on spectrophotometry^39,40^.

### Cells and virus

NiV (strain Bangladesh/200401066, Genbank ID AY988601) was kindly provided by the Special Pathogens Branch of the Centers for Disease Control and Prevention, Atlanta, Georgia, United States. The stock used in this study was passage 3 from a throat swab collected from patient #3001 (10-year-old male) in Bangladesh on January 22, 2004^41^. All virus propagation was performed in VeroE6 cells cultured in Dulbecco’s modified Eagle’s medium (DMEM, Sigma) supplemented with 2% fetal bovine serum (Gibco), 1 mM L-glutamine (Gibco), 50 U/ml penicillin (Gibco), and 50 μg/ml streptomycin (Gibco) (2% DMEM). VeroE6 cells were maintained in DMEM supplemented with 10% fetal calf serum, 1 mM L-glutamine, 50 U/ml penicillin, and 50 μg/ml streptomycin.

### Virus titration

Virus titrations were performed by endpoint titration in VeroE6 cells, which were inoculated with tenfold serial dilutions of virus swab media or tissue homogenates. After 1 h incubation at 37 °C and 5% CO_2_, tissue homogenate dilutions were removed, washed twice with PBS and replaced with 100 μl 2% DMEM. Cytopathic effect was scored at 5 DPI and the TCID_50_ was calculated from a minimum of 4 replicates by the Spearman-Karber method^42^.

### RNA extraction and quantitative reverse-transcription polymerase chain reaction

RNA was extracted from throat and nose swabs using a QiaAmp Viral RNA kit (Qiagen) according to the manufacturer’s instructions. RNA (5 μl) was tested with the Rotor-Gene probe kit (Qiagen) according to instructions of the manufacturer, using a N gene specific assay^43^. Dilutions of NiV standards with known TCID50 values were run in parallel.

### Virus neutralization

Heat-inactivated sera (30 min, 56 °C) were serially diluted (2x) in 2% DMEM. Hereafter, 100 TCID_50_ of NiV was added. After 1 hr of incubation at 37 °C and 5% CO_2_, serum:virus mixture was added to VeroE6 cells and incubated at 37 °C and 5% CO_2_. At 5 DPI, cytopathic effect was scored. The virus neutralization titer was expressed as the reciprocal value of the highest dilution of the serum which still inhibited virus replication.

### Production NiV-G and -F proteins

Nipah proteins^15^ were produced as follows: NiV-G Malaysia (95.7% identical to NiV-G Bangladesh (amino acids), residues E144–T602, gene accession number NC_002728) was cloned into the pHLSEC mammalian expression vector^44^ and NiV-F Malaysia (98.5% identical to NiV-F Bangladesh (amino acids), residues G26–D482, gene accession number AY816748.1) was cloned into the pHLSEC vector containing a C-terminal GCNt trimerization motif^45^. The constructs were transiently expressed in human embryonic kidney (HEK) 293 T cells. Supernatant was diafiltrated using the AKTA FLUX system (GE Healthcare) against either PBS, pH 7.4 (NiV-G) or buffer containing 10 mM Tris and 150 mM NaCl, pH 8.0 (NiV-F). The proteins were further purified by Ni-NTA immobilized metal-affinity chromatography using His-Trap HP columns (GE Healthcare) followed by size exclusion chromatography. NiV-G was purified using a Superdex 200 10/300 Increase GL column (GE healthcare) equilibrated in PBS pH 7.4 and NiV-F was purified using a Superose 6 Increase 10/300 GL column (GE healthcare) equilibrated in 10 mM Tris and 150 mM NaCl pH 8.0.

### Enzyme-linked immunosorbent assay for Nipah G, N, and F proteins

Maxisorp plates (Nunc) were coated overnight at 4 °C with 5 µg of G, N (NiV Malaysia, Native Antigen Company) or F protein per plate in Carb/Bicarb binding buffer (4.41 g KHCO_3_ and 0.75 g Na_2_CO_3_ in 1 L distilled water). After blocking with 5% milk in PBS with 0.01% tween (PBST), serum in 5% milk in PBST was incubated at RT for 1 hr. Antibodies were detected using affinity-purified antibody peroxidase-labeled goat-anti-monkey IgG (Seracare) in 5% milk in PBST and TMB 2-component peroxidase substrate (Seracare) and read at 450 nm. All wells were washed 3x with PBST in between steps.

### ELISpot assay and ICS analysis

PBMCs were isolated from ethylene diamine tetraaceticacid (EDTA) whole blood using LeucosepTM tubes (Greiner Bio-one International GmbH) and Histopaque®-1077 density gradient cell separation medium (Sigma-Aldrich) according to the manufacturers’ instructions. The ImmunoSpot® Human IFN-γ Single-Color Enzymatic ELISpot Assay Kit was utilized according to the manufacturer’s protocol (Cellular Technology Limited). PBMCs were plated at a concentration of 300,000 cells per well and were stimulated with six contiguous peptide pools spanning the length of the G protein sequence at a concentration of 2 µg/mL per peptide. One peptide (sequence AFNTVIALLGSIVII) was excluded due to false positive results. Analysis was performed using the CTL ImmunoSpot® Analyzer and ImmunoSpot® Software (Cellular Technology Limited). Spot forming units (SFU) per 1.0 × 10^6^ PBMCs were summed across the 6 peptide pools for each animal.

### Histology and in situ hybridization

Harvested tissues were fixed for a minimum of 7 days in 10% neutral-buffered formalin and subsequently embedded in paraffin. Hematoxylin and eosin (H&E) staining, in situ hybridization (ISH), and immunohistochemistry were performed on tissue sections and cell blocks. Detection of NiV viral RNA was performed using the RNAscope FFPE assay (Advanced Cell Diagnostics Inc., Newark, USA) as previously described^46^ and in accordance with the manufacturer’s instructions. Briefly, tissue sections were deparaffinized and pretreated with heat and protease before hybridization with target-specific probes for NiV. Ubiquitin C and the bacterial gene, dapB, were used as positive and negative controls, respectively. Whole-tissue sections for selected cases were stained for NiV RNA, UBC, and dapB by the RNAscope VS FFPE assay (RNAscopeVS, Newark, USA) using the Ventana Discovery XT slide autostaining system (Ventana Medical Systems Inc., Tucson, USA). Detection of NiV viral antigen (whole N protein or antibody against peptide CQETSAGRQESNVQA) was detected using a polyclonal rabbit antibody (Genscript) at a 1:500 dilution. The immunohistochemistry assay was carried out on a Discovery ULTRA automated-staining instrument (Roche Tissue Diagnostics) with a Discovery ChromoMap DAB (Ventana Medical Systems) kit. A board-certified veterinary anatomic pathologist blinded to the study groups evaluated all tissue slides. Histology score was determined by scoring 6 lung lobes for each animal for the following characteristics: lymphoid cuffing; pneumonia, bronchointerstitial, with fibrin, edema and syncytial cells; and perivascular and alveolar edema and fibrin. Scoring was as follows: 0 = no lesions; 1 = 1–10%; 2 = 11–25%; 3 = 26–50%; 4 = 51–75%; 5 = 76–100%. All scores per animal were added to allow a maximum score of 90.

### Statistical analysis

Kruskal–Wallis test for multiple comparisons was used to test for statistical significance. *P*-values < 0.05 were considered significant.

### Reporting summary

Further information on research design is available in the Nature Research Reporting Summary linked to this article.

## Supplementary information

Supplementary file

REPORTING SUMMARY

## Data Availability

All raw data are available on Figshare: 10.6084/m9.figshare.20161934.

## References

[1] Chua KB (2000). Nipah virus: a recently emergent deadly paramyxovirus. Science.

[2] McKee, C. D. et al. The ecology of Nipah Virus in Bangladesh: a nexus of land-use change and opportunistic feeding behavior in bats. *Viruses* **13**, 169 (2021). 10.3390/v13020169 PMC7910977 33498685

[3] ProMED. *PRO/AH/EDR> Nipah virus—Bangladesh (02) 20200203.6950171*. https://promedmail.org/promed-post/?id=20200203.6950171 (2020).

[4] Arunkumar G (2019). Outbreak investigation of Nipah virus disease in Kerala, India, 2018. J. Infect. Dis..

[5] Gurley ES (2007). Person-to-person transmission of Nipah virus in a Bangladeshi community. Emerg. Infect. Dis..

[6] Nikolay B (2019). Transmission of Nipah Virus—14 years of investigations in Bangladesh. N. Engl. J. Med..

[7] Weatherman S, Feldmann H, de Wit E (2018). Transmission of henipaviruses. Curr. Opin. Virol..

[8] World Health Organization. *R&D Blueprint and Nipah*. https://www.who.int/teams/blueprint/nipah (2019).

[9] Amaya M, Broder CC (2020). Vaccines to emerging viruses: Nipah and Hendra. Annu. Rev. Virol..

[10] Broder CC, Weir DL, Reid PA (2016). Hendra virus and Nipah virus animal vaccines. Vaccine.

[11] Geisbert TW (2021). A single dose investigational subunit vaccine for human use against Nipah virus and Hendra virus. npj Vaccines.

[12] Voysey, M. et al. Safety and efficacy of the ChAdOx1 nCoV-19 vaccine (AZD1222) against SARS-CoV-2: an interim analysis of four randomised controlled trials in Brazil, South Africa, and the UK. *Lancet*10.1016/S0140-6736(20)32661-1 (2020). 10.1016/S0140-6736(20)32661-1 PMC7723445 33306989

[13] Falsey AR (2021). Phase 3 safety and efficacy of AZD1222 (ChAdOx1 nCoV-19) Covid-19 vaccine. N. Engl. J. Med..

[14] Voysey M (2021). Single-dose administration and the influence of the timing of the booster dose on immunogenicity and efficacy of ChAdOx1 nCoV-19 (AZD1222) vaccine: a pooled analysis of four randomised trials. Lancet.

[15] van Doremalen N (2019). A single-dose ChAdOx1-vectored vaccine provides complete protection against Nipah Bangladesh and Malaysia in Syrian golden hamsters. PLoS Negl. Trop. Dis..

[16] Brining DL (2010). Thoracic radiography as a refinement methodology for the study of H1N1 influenza in cynomologus macaques (Macaca fascicularis). Comp. Med..

[17] Bossart KN (2012). A Hendra Virus G glycoprotein subunit vaccine protects african green monkeys from Nipah virus challenge. Sci. Transl. Med..

[18] Lo MK (2014). Single-dose replication-defective VSV-based Nipah virus vaccines provide protection from lethal challenge in Syrian hamsters. Antivir. Res..

[19] Prescott J (2015). Single-dose live-attenuated vesicular stomatitis virus-based vaccine protects African green monkeys from Nipah virus disease. Vaccine.

[20] Price, A et al. *Nipah virus assays and animal models for vaccine development*. https://media.tghn.org/medialibrary/2021/02/Nipah_Virus_Assays_and_Animal_Models_for_Vaccine_Development_final.pdf (2021).

[21] Snoy PJ (2010). Establishing efficacy of human products using animals: The US Food and Drug Administration’s “Animal Rule”. Vet. Pathol..

[22] U.S. Food & Drug. *Animal Rule Approvals*. https://www.fda.gov/drugs/nda-and-bla-approvals/animal-rule-approvals (2021).

[23] Flaxman A (2021). Reactogenicity and immunogenicity after a late second dose or a third dose of ChAdOx1 nCoV-19 in the UK: a substudy of two randomised controlled trials (COV001 and COV002). Lancet.

[24] Wong KT (2002). Nipah virus infection. Am. J. Pathol..

[25] Dawes BE, Freiberg AN (2019). Henipavirus infection of the central nervous system. Pathog. Dis..

[26] Tan CT (2002). Relapsed and late-onset Nipah encephalitis. Ann. Neurol..

[27] Lee JH (2020). The use of large-particle aerosol exposure to Nipah virus to mimic human neurological disease manifestations in the African Green monkey. J. Infect. Dis..

[28] Mire CE (2014). A recombinant Hendra Virus G glycoprotein subunit vaccine protects nonhuman primates against Hendra virus challenge. J. Virol..

[29] Yoneda M (2013). Recombinant Measles virus vaccine expressing the Nipah virus glycoprotein protects against lethal Nipah virus challenge. PLoS ONE.

[30] Mire CE (2019). Use of single-injection recombinant vesicular Stomatitis virus vaccine to protect nonhuman primates against lethal Nipah virus disease. Emerg. Infect. Dis..

[31] ClinicalTrials.gov. *A Phase 1 Study to Evaluate Safety & Immunogenicity of rVSV-Nipah Virus Vaccine Candidate PHV02 in Healthy Adult Subjects*. https://clinicaltrials.gov/ct2/show/NCT05178901 (2022).

[32] ClinicalTrials.gov. *Safety and Immunogenicity of a Nipah Virus Vaccine*. https://clinicaltrials.gov/ct2/show/NCT04199169 (2022).

[33] van Doremalen N (2020). A single dose of ChAdOx1 MERS provides protective immunity in rhesus macaques. Sci. Adv..

[34] van Doremalen, N. et al. ChAdOx1 nCoV-19 vaccine prevents SARS-CoV-2 pneumonia in rhesus macaques. *Nature*10.1038/s41586-020-2608-y (2020). 10.1038/s41586-020-03099-2 33469217

[35] Feldmann F, Shupert WL, Haddock E, Twardoski B, Feldmann H (2019). Gamma irradiation as an effective method for inactivation of emerging viral pathogens. Am. J. Trop. Med. Hyg..

[36] Haddock E, Feldmann F, Feldmann H (2016). Effective chemical inactivation of Ebola virus. Emerg. Infect. Dis..

[37] Munster VJ (2020). Respiratory disease in rhesus macaques inoculated with SARS-CoV-2. Nature.

[38] Dicks MDJ (2012). A novel Chimpanzee adenovirus vector with low human seroprevalence: improved systems for vector derivation and comparative immunogenicity. PLoS ONE.

[39] Bewig B, Schmidt WE (2000). Accelerated titering of adenoviruses. BioTechniques.

[40] Maizel JV, White DO, Scharff MD (1968). The polypeptides of adenovirus. Virology.

[41] de Wit E (2014). Foodborne Transmission of Nipah Virus in Syrian Hamsters. PLoS Pathog..

[42] Kärber G (1931). Beitrag zur kollektiven Behandlung pharmakologischer Reihenversuche. Arch. f. Exp. Pathol. u. Pharmakol..

[43] DeBuysscher BL (2013). Comparison of the pathogenicity of Nipah virus isolates from Bangladesh and Malaysia in the Syrian Hamster. PLoS Negl. Trop. Dis..

[44] Aricescu AR, Lu W, Jones EY (2006). A time- and cost-efficient system for high-level protein production in mammalian cells. Acta Crystallogr. D. Biol. Crystallogr..

[45] Chan Y-P (2012). Biochemical, conformational, and immunogenic analysis of soluble trimeric forms of Henipavirus fusion glycoproteins. J. Virol..

[46] Wang F (2012). RNAscope. J. Mol. Diagn..

