# Supplementary material for: ChAdOx1 NiV vaccination protects against lethal Nipah Bangladesh virus infection in African green monkeys
Source: NPJ Vaccines. 2022 Dec 21;7:171. doi: 10.1038/s41541-022-00592-9 (PMC9768398; doi:10.1038/s41541-022-00592-9)
Supplement: Supplementary file 1 — Supplementary file [file 41541_2022_592_MOESM1_ESM.pdf]

Treatment	Animal	Day 1	Day 2	Day 3	Day 4	Day 5	Day 6	Day 7
Prime-Boost ChAdOx1 NiV	1	RA, 3	RA, 3	RA, 3	RA, 3	RA, 3	RA, 5	RA, 5
	2	RA, 5	RA, 5	RA, 5	RA, 5	RA, 3	RA, 5	RA, 3
	3	RA, 5	RA, 5	RA, 5	RA, 5	RA, 3	RA, 5	RA, 5
	4	RA, 5	RA, 5	RA, 5	RA, 3	RA, 3	RA, 3	RA, 3
Prime ChAdOx1 NiV	5	RA, 3	RA, 8	RA, 8	RA, 3	RA, 3	RA, 5	RA, 5
	6	RA, 3	RA, 10	RA, 10	RA, 5	RA, 5	RA, 5	RA, 3
	7	RA, 3	RA, 5	RA, 3	RA, 5	RA, 3	RA, 5	RA, 5
	8	RA, 5	RA, 5	RA, 5	RA, 3	RA, 5	RA, 5	RA, 5

Prime-Boost ChAdOx1 GFP	9	RA, RF, 3	RA, RF, 5	RA, RF, 3	RA, RF, 8	RA, RF, 5	RA, HP, RF, ND, IR 25	RA, HP, RF, 40<br>Fever 40.3°C, ND, IR, OM, CY
	10	RA,5	RA, 5	-, 0	RA, IR, 8	IR,5	RA, RF, IR, NS, 25	HP, RF, RA, IR, NS, 35
	11	RA, 5	RA, 5	RA, 5	RA, IR,10	HP, RA, IR,NS ,40	n/a	
	12	RA,5	RA,5	RA, IR,10	RA, RF, IR,15	RA, RF, IR,15	RA, RF, HP, ND, IR, OM CY, 40	n/a

Supplementary Table 1. Clinical signs in AGMs inoculated with NiV-B. RA = reduced appetite; RF = ruffled fur; HP = hunched posture; ND= nasal discharge; IR= increased respirations, OM= open mouth breathing, CY= cyanotic, NS= neurological symptoms, # = total clinical score

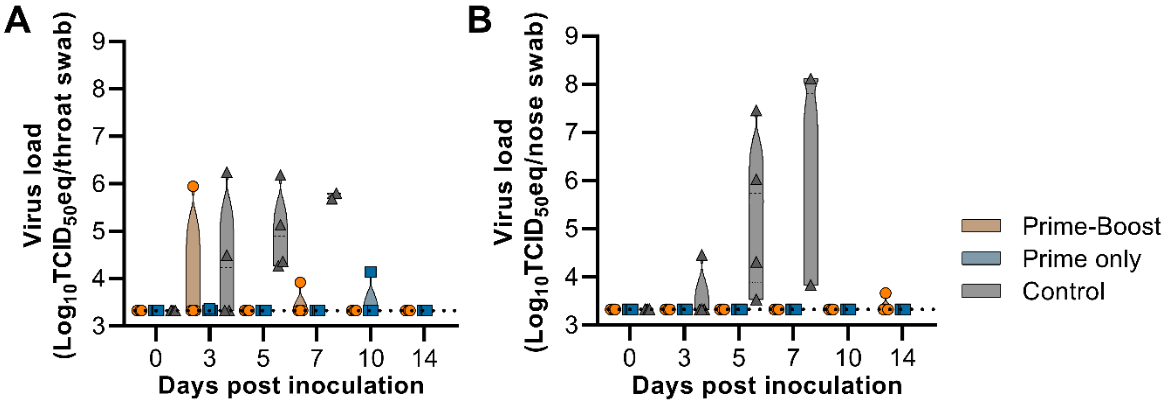

Supplementary Figure 1. Viral load in throat and nose swabs. Truncated violin plots of NiV genome copies in throat swabs (A) and nose swabs (B). At 7 DPI, only 2 control animals were part of the study. Dotted line indicates limit of quantification. For all panels, orange indicates prime-boost vaccinated animals, blue indicates prime only vaccinated animals, and grey indicates control animals.

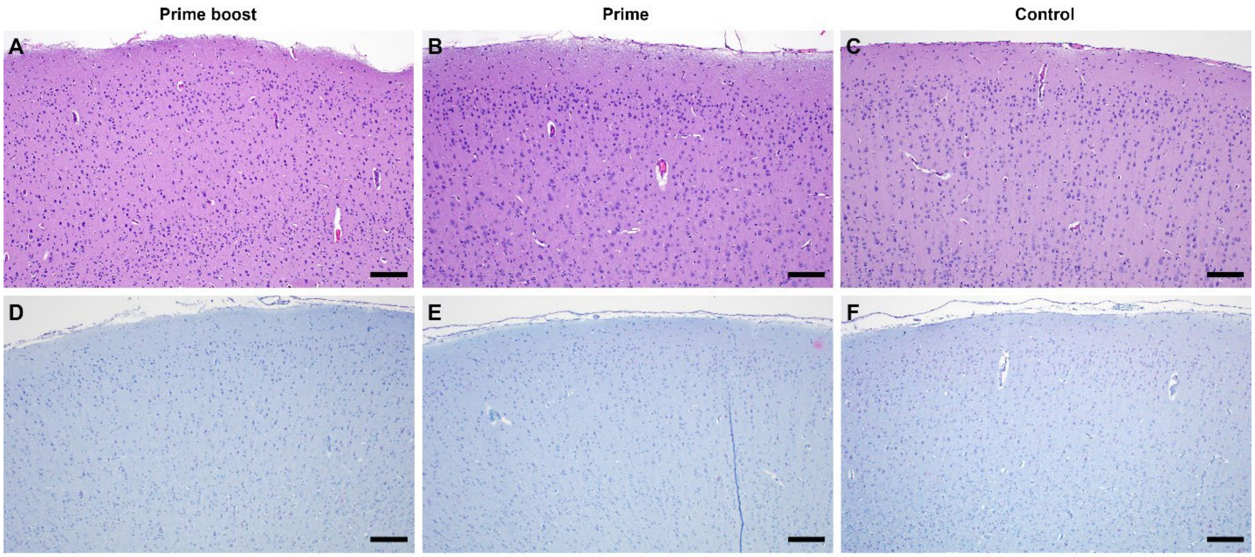

Supplementary Figure 2. Lack of NiV infection of brain tissue in AGMs. (A-C) Cerebrum tissue sections were stained with hematoxylin and eosin. No pathology was observed. **(D-F)**

Immunohistochemistry for NiV N protein, resulting in a brown stain. No immunoreactivity was observed. Magnification: 100x; bar = 50µm.

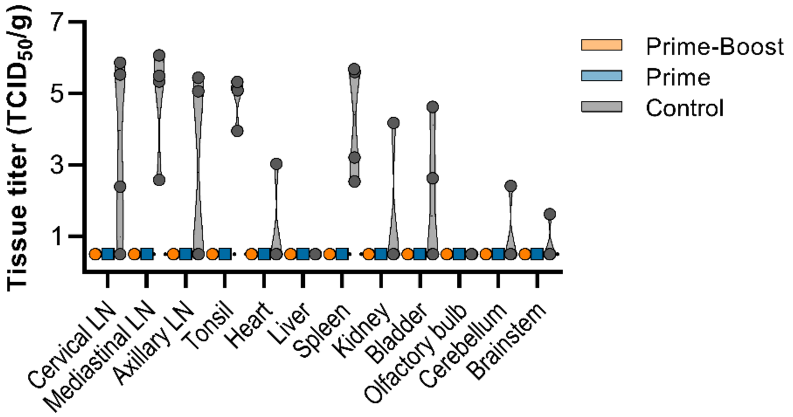

Supplementary Figure 3. Presence of infectious virus in non-respiratory or brain tissue of African green monkeys inoculated with Nipah virus. Violin plot of infectious virus detected in respiratory tract and brain tissue. Orange circles, prime-boost vaccine; blue squares, prime–only vaccine; grey triangles, controls. No statistical tests were performed since samples were obtained on different days.
