# Supplementary material for: ChAdOx1 NiV vaccination protects against lethal Nipah Bangladesh virus infection in African green monkeys
Source: NPJ Vaccines. 2022 Dec 21;7:171. doi: 10.1038/s41541-022-00592-9 (PMC9768398; doi:10.1038/s41541-022-00592-9)
Supplement: Supplementary file 2 — REPORTING SUMMARY [file 41541_2022_592_MOESM2_ESM.pdf]

nature portfolio

Corresponding author(s): Vincent J Munster

Last updated by author(s): Oct 20, 2022

Reporting Summary

Nature Portfolio wishes to improve the reproducibility of the work that we publish. This form provides structure for consistency and transparency in reporting. For further information on Nature Portfolio policies, see our Editorial Policies and the Editorial Policy Checklist.

Statistics

For all statistical analyses, confirm that the following items are present in the figure legend, table legend, main text, or Methods section.

n/a	Confirmed	
☐	☒	The exact sample size (*n*) for each experimental group/condition, given as a discrete number and unit of measurement
☒	☐	A statement on whether measurements were taken from distinct samples or whether the same sample was measured repeatedly
☐	☒	The statistical test(s) used AND whether they are one- or two-sided<br>*Only common tests should be described solely by name; describe more complex techniques in the Methods section.*
☐	☒	A description of all covariates tested
☐	☒	A description of any assumptions or corrections, such as tests of normality and adjustment for multiple comparisons
☐	☒	A full description of the statistical parameters including central tendency (e.g. means) or other basic estimates (e.g. regression coefficient) AND variation (e.g. standard deviation) or associated estimates of uncertainty (e.g. confidence intervals)
☐	☒	For null hypothesis testing, the test statistic (e.g. *F*, *t*, *r*) with confidence intervals, effect sizes, degrees of freedom and *P* value noted<br>*Give P values as exact values whenever suitable.*
☒	☐	For Bayesian analysis, information on the choice of priors and Markov chain Monte Carlo settings
☒	☐	For hierarchical and complex designs, identification of the appropriate level for tests and full reporting of outcomes
☒	☐	Estimates of effect sizes (e.g. Cohen's *d*, Pearson's *r*), indicating how they were calculated

Our web collection on statistics for biologists contains articles on many of the points above.

Software and code

Policy information about availability of computer code

Data collection	*Provide a description of all commercial, open source and custom code used to collect the data in this study, specifying the version used OR state that no software was used.*
Data analysis	Data were analyzed using Graphpad Prism 8.2.1, and ImmunoSpot software 7.0 11.0 Professional Analyzer DC

For manuscripts utilizing custom algorithms or software that are central to the research but not yet described in published literature, software must be made available to editors and reviewers. We strongly encourage code deposition in a community repository (e.g. GitHub). See the Nature Portfolio guidelines for submitting code & software for further information.

Data

Policy information about availability of data

All manuscripts must include a data availability statement. This statement should provide the following information, where applicable:

- Accession codes, unique identifiers, or web links for publicly available datasets
- A description of any restrictions on data availability
- For clinical datasets or third party data, please ensure that the statement adheres to our policy

The processed data are available at Figshare (https://doi.org/10.6084/m9.figshare.20161934.v2) and are provided in the Source Data file.

Field-specific reporting

Please select the one below that is the best fit for your research. If you are not sure, read the appropriate sections before making your selection.

☒ Life sciences ☐ Behavioural & social sciences ☐ Ecological, evolutionary & environmental sciences

For a reference copy of the document with all sections, see nature.com/documents/nr-reporting-summary-flat.pdf

Life sciences study design

All studies must disclose on these points even when the disclosure is negative.

Sample size	Sample size was determined based on previous experience measuring survival in African green monkeys (100% lethal without treatment) and minimum animal numbers required if vaccine is 100% protective (Fisher exact test, N=4).
Data exclusions	No data were excluded.
Replication	Lung histology: for each animal (n=4), 3 sections were evaluated from all 6 lung lobes.<br>Serological analysis: Serum samples were analyzed in duplicate from each animal<br>Virological analysis: All samples were titrated in quadruplicate<br>All replications were successful.
Randomization	Animals were randomly assigned to groups
Blinding	Blinding was done for the following personnel:<br>- Veterinary pathologists reviewing histology<br>- Technician analyzing virology data<br>- Technicians analyzing humoral response<br>- Technician weighing animals

Reporting for specific materials, systems and methods

We require information from authors about some types of materials, experimental systems and methods used in many studies. Here, indicate whether each material, system or method listed is relevant to your study. If you are not sure if a list item applies to your research, read the appropriate section before selecting a response.

Materials & experimental systems

n/a	Involved in the study	
☐	☒	Antibodies
☐	☒	Eukaryotic cell lines
☒	☐	Palaeontology and archaeology
☐	☒	Animals and other organisms
☒	☐	Human research participants
☒	☐	Clinical data
☒	☐	Dual use research of concern

Methods

n/a	Involved in the study	
☒	☐	ChIP-seq
☒	☐	Flow cytometry
☒	☐	MRI-based neuroimaging

Antibodies

Antibodies used	Affinity-purified antibody peroxidase-labeled goat-anti-monkey IgG (Seracare) 1:1000
Validation	Validated by company

Eukaryotic cell lines

Policy information about cell lines

Cell line source(s)	VeroE6 in-house for >10 years
Authentication	Vero E6: Cytochrome B authenticated in-house
Mycoplasma contamination	Vero E6: Mycoplasma testing confirmed negative monthly

Commonly misidentified lines (See ICLAC register)	No commonly misidentified cell lines were used.

Animals and other organisms

Policy information about studies involving animals; ARRIVE guidelines recommended for reporting animal research

Laboratory animals	African green monkeys, adults
Wild animals	No wild animals were used.
Field-collected samples	No samples were collected in the field.
Ethics oversight	All animal experiments were approved by the Institutional Animal Care and Use Committee of Rocky Mountain Laboratories, NIH and carried out by certified staff in an Association for Assessment and Accreditation of Laboratory Animal Care (AAALAC) International accredited facility, according to the institution's guidelines for animal use, following the guidelines and basic principles in the NIH Guide for the Care and Use of Laboratory Animals, the Animal Welfare Act, United States Department of Agriculture and the United States Public Health Service Policy on Humane Care and Use of Laboratory Animals.

Note that full information on the approval of the study protocol must also be provided in the manuscript.
